# Virus-host interaction mechanisms in interferon therapy for hepatitis B virus infection: recent advances

**DOI:** 10.3389/fimmu.2025.1603544

**Published:** 2025-06-27

**Authors:** Jiebing Zhang, Tao Lou, Minmin Zhu, Chengkang Wang, Kai Gong, Yingping Wu

**Affiliations:** ^1^ Department of Infectious Diseases, the Fourth Affiliated Hospital of School of Medicine, and International School of Medicine, International Institutes of Medicine, Zhejiang University, Yiwu, China; ^2^ Department of Clinical Laboratory, the Fourth Affiliated Hospital of School of Medicine, and International School of Medicine, International Institutes of Medicine, Zhejiang University, Yiwu, China

**Keywords:** interferon-α, chronic hepatitis b, antiviral treatment, host factors, virus-host interactions

## Abstract

Chronic hepatitis B virus (HBV) infection has been implicated in the development of liver diseases, such as hepatitis, fibrosis, cirrhosis, and cancer, which negatively affect the patients’ quality of life and impacts a high economic strain on patients. The persistence of covalently closed circular DNA (cccDNA) allows the propagation of the infection, and no drug have been developed to completely eliminate cccDNA. The available drugs for chronic hepatitis B (CHB) are classified into nucleos(t)ide analogs (NAs) and interferon-α (IFN-α)/pegylated interferon α (Peg-IFN-α). However, these treatments do not effectively eradicate hepatitis B surface antigen (HBsAg) and their clinical efficacy is limited. The potential of IFN-based clinical cure is increasingly attracting interest from hepatologists, but the therapeutic outcomes of this intervention are suboptimal and some of them are associated with various complications. Although several novel antiviral drugs are being investigated, however, achieving a clinical cure based on monotherapy is currently challenging. The efficacy of IFN therapy is influenced by host and viral factors. This article provides a comprehensive review of host-related factors that affect the IFN therapy for CHB. A thorough understanding and management of these host-related factors will enhance the efficacy of interferon treatment, minimize adverse reactions, improve patient tolerance, and thereby increasing the clinical cure rate of hepatitis B.

## 1 Introduction

Hepatitis B virus infection represents a significant global public health challenge, with the potential to progress into chronic viral hepatitis B, liver fibrosis, liver cirrhosis, and ultimately hepatocellular carcinoma (HCC), thereby posing a substantial threat to human health. Data from the World Health Organization’s (WHO’s) 2024 Global Hepatitis Report show that about 254 million people lived with viral hepatitis B in 2022. Enhanced data from 187 countries showed that the mortality associated with viral hepatitis rose from 1.1 million in 2019 to 1.3 million in 2022, with hepatitis B responsible for 83% of these deaths. This suggests that the incidence of hepatitis-related cancers and associated fatalities have been on the rise (1). An effective way to avoid HBV infection is to get vaccinated against HBV. WHO recommends that all children receive a monovalent dose of hepatitis B vaccine within the first 24 hours after birth followed by an additional 2–3 doses in infancy (3), however, there are still many countries that do not have full vaccination coverage.

Antiviral therapy is generally not necessary in patients with symptomatic acute hepatitis B (AHB) because >95% of immunocompetent adults with acute hepatitis B recover spontaneously (2). During the onset of AHB children, appropriate drugs can be selected for antiviral therapy according to age, so as to promote clinical cure and reduce the risk of chronicity (3). The major treatments for CHB are NAs and IFN-α/Peg-IFN-α. However, neither monotherapy nor combination therapy have satisfactory outcomes. The mechanisms of NAs involve the inhibition of the reverse transcription of pregenomic RNA (pgRNA) into relaxed circular DNA (rcDNA) by decreasing the reverse transcriptase activity. Nonetheless, these agents do not directly target the formation of cccDNA (4). Consequently, most patients require prolonged NA therapy, and the rate of virologic recurrence is high after drug cessation (5). Peg-IFN-α exerts its therapeutic effects by directly act against HBV infection in hepatocytes and by modulating immune responses (6). Compared to NAs, the duration of IFN therapy is shorter and elicits a more robust and durable serologic response (7). However, IFN therapy alone is effective only in a subset of patients and is associated with relatively poor tolerability. For those who show good response to IFN therapy may sustain a virologic response after discontinuation, but overall efficacy is poor (8).

**Figure 1:**
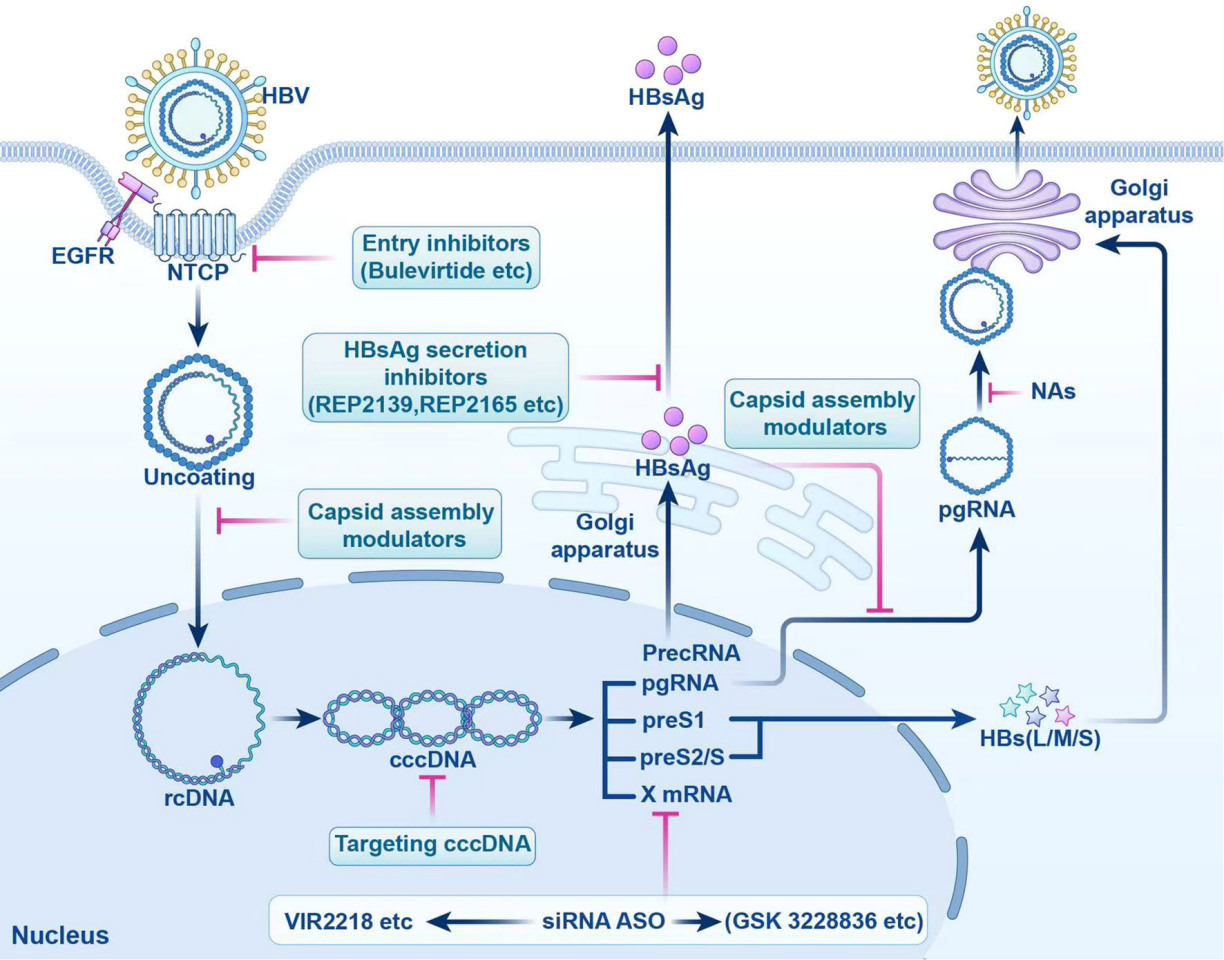
Targeted Drugs in the HBV Life cycle. This figure shows the action sites of entry inhibitors. cccDNA, targeted covalently closed circular DNA; siRNA, small interfering RNA; ASO, antisense oligonucleotides; HBV, hepatitis B virus; NTCP, sodium taurocholate cotransporter peptide; EGFR, epidermal growth factor receptor; rcDNA, relaxed circular DNA; HBsAg, hepatitis B surface antigen; PrecRNA, precore mRNA; pgRNA, pregenomic RNA; preS1, presurface mRNA 1; preS2/S, presurface mRNA2/S; HBs(L/M/S), hepatitis B surface antigen (large/medium/small); NAs, nucleos(t)ide analogs.

**Figure 2:**
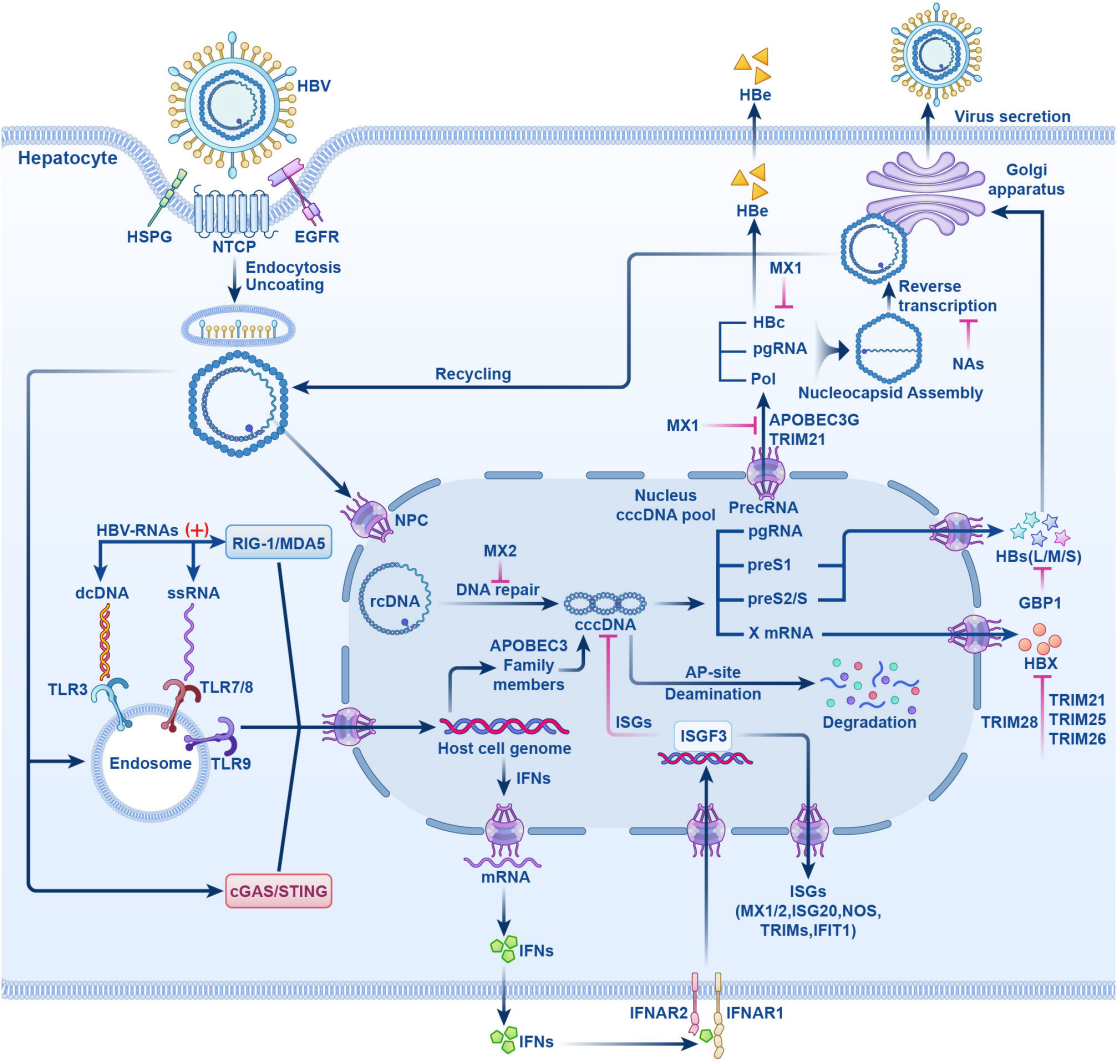
The HBV life cycle and the mechanisms of action of NAs and IFN-α. HBV induces endocytosis by binding to NTCP receptor on hepatocytes, it also can bind to HSPG and EGFR and enter liver cells. After uncoating, rcDNA enters the NPC, repaired by host enzymes in the nucleus to form cccDNA. cccDNA is transcribed by host polymerases into the suite of viral RNAs that are translated into all viral proteins that are required for replication. Pregenomic RNA(pgRNA) is transcribed from cccDNA. HBc, pol and pgRNA are assembled and reverse transcribed to form rcDNA. Finally, rcDNA binds to surface antigens to form complete HBV particles and secretes. Partial rcDNA can circulate to the nucleus to increase the number of viruses. Upon viral entry into the host cells, viral nucleic acid sensor proteins, including cGAS/STING, TLRs -3, -7, -8, and −9, or RIG-I/MDA5, recognize viral DNA or RNA and generate IFNs. IFNs exert antiviral effects by inducing the host to produce IFN stimulated genes (ISGs). NTCP, sodium taurocholate cotransporter peptide; HSPG, hepatocyte-associated heparan sulfate proteoglycans; EGFR, epidermal growth factor receptor; rcDNA, relaxed circular DNA; NPC, nuclear pore complex; cccDNA, form covalently closed circular DNA; pol, polymerase; cGAS/STING, cyclic GMP-AMP synthase/stimulator of interferon genes; TLRs, Toll-like receptors; RIG-I/MDA5, retinoic acid-inducible gene I/melanoma differentiation-associated protein 5; ISGs, IFN stimulated genes; (+) stands for “activation”; HBs(L/M/S), HBV surface antigen (large, middle, small); HBc, HBV core antigen; HBx, HBV x protein; HBe, HBV e antigen; NAs, nucleotide analogs; MX1/2, myxovirus resistance proteins1/2; APOBEC3, apolipoprotein B mRNA editing enzyme catalytic polypeptide-like 3; ISGF3, IFN-stimulating gene factor 3; IFNAR1/2, interferon-α receptor I/II; TRIM, tripartite motif protein; NOS, nitric oxide synthase; GBP1, guanylate binding protein1; IFIT1, IFN-induced tetratricopeptide repeat 1.

Currently, research and development strategies for antiviral drugs are primarily categorized into two approaches: one targets the HBV life cycle, including entry inhibitors, capsid assembly modulators (CAM), HBsAg secretion inhibitors, cccDNA silencers, small interfering RNAs, and antisense oligonucleotides (**Figure 1**); the other targets the host immune system, comprising therapeutic vaccines, monoclonal antibodies, apoptosis inducers, innate immune activators such as Toll-like receptor (TLR) agonists, programmed cell death protein 1 (PD-1)/programmed cell death 1 ligand 1 (PD-L1) inhibitors, and HBV-specific immune reconstitution strategies (e.g., T-cell immunomodulators) (9). Among them, CAM can act at multiple steps that inhibit the early and late steps of the viral life cycle, including assembly of aberrant or empty capsids (thereby inhibiting HBV DNA replication) and interference with disassembly of incoming virions and intracellular recycling of capsids (thereby inhibiting cccDNA establishment and replenishment) (10–12). Although several drugs are being tested in clinical trials, no new antiviral drugs have been approved for clinical use, and IFN is still the most promising agent for treating CHB. In this article, we review the life cycle of HBV (**Figure 2**), the antiviral therapy mechanism of IFN and NAs (**Figure 2**), the limitations of current antiviral therapies, and summarize some host-related factors that potentially influence the effect of IFN anti-hepatitis B virus therapy.

## 2 Molecular mechanisms of HBV infection

HBV is a hepatotropic DNA virus with a genome that consists of partially double-stranded circular DNA. This genome encodes several proteins, including the HBsAg, hepatitis B core antigen (HBcAg), hepatitis B e antigen (HBeAg), viral polymerase, and the hepatitis B x(HBx)protein (13). HBV virions infect hepatocytes and primarily enter through high-affinity binding between viral surface proteins on infectious virions and sodium taurocholate cotransporter peptide (NTCP) on the surface of hepatocytes. Heparan sulfate proteoglycans (HSPG), epidermal growth factor receptor (EGFR), and neuropilin-1 (NRP1) on the hepatocyte surface also act as cofactors to promote HBV entry into hepatocytes (14–17). The interaction between viruses and receptors is internalized through endothelial cell proliferation, the incoming nucleocapsid in the cytoplasm is directed to the nucleus along with the microtubules (18–20). After uncoating at the nuclear pore, the rcDNA genome is released into the nucleus, which utilizes host enzymes to convert into cccDNA. This cccDNA associates with histones to form a stable episomal template, from which viral RNA is transcribed (16). Using cccDNA as the transcription template, five viral transcripts are synthesized from distinct transcription start sites: the 3.5~3.6 kb precore mRNA, the 3.5 kb pregenomic RNA(pgRNA), the 2.4 and 2.1 kb surface mRNA, and the 0.7 kb X mRNA. The pgRNA can act as a template for the translation of viral core protein and polymerase but also as a template for reverse transcription (21). Once cccDNA is established in the nucleus, it is challenging to eradicate from infected cells, and it can persist until the host cell undergoes apoptosis, contributing significantly to the pathogenesis of chronic infection (22).

HBV does not directly cause cellular damage; rather, the inflammation and necrosis of liver tissue primarily result from the host’s immune response to invading antigens, activating the immune system to target and destroy infected liver cells. This immune-mediated liver injury can lead to liver fibrosis and HCC during chronic HBV infection (23). During acute HBV infection, virus-specific CD8^+^ T cells are essential for controlling and eradicating the infection (24). Chronic HBV infection is characterized by a diminished or undetectable HBV-specific CD8^+^ T cell response, alongside exhausted HBV-specific CD8^+^ T cells incapable of virus clearance (25). In individuals with CHB, phenotypic alterations and functional impairments of natural killer (NK) cells have been observed (26). NK cells contribute to this process by secreting IFN-γ or tumor necrosis factor-alpha (TNF-α) to induce apoptosis in infected cells, or by directly eliminating these cells through the expression of granzymes, Fas ligand, or killer cell immunoglobulin-like receptors (26). IFN-γ and TNF-α production in NK cells are also strongly inhibited during chronic HBV infection (27).

## 3 CHB treatment drugs

Effective management of CHB is essential for reducing the risk of HBV-related liver complications, including hepatitis, cirrhosis, liver failure, and HCC. The spontaneous clearance of HBsAg is infrequent, with an annual incidence rate of approximately 1% (28). Current guidelines for the diagnosis and treatment of CHB, as outlined by the European Association for the Study of the Liver, the American Association for the Study of Liver Diseases, the Chinese Society of Hepatology, and the Chinese Society of Infectious Diseases, recommend the use of IFN-α/peg-IFN-α and NAs in the treatment of patients with HBV infection, tailored to the specific needs of the patient (2, 13, 29).

### 3.1 NAs

The frequently used NAs for the treatment of HBV are Entecavir (ETV), Tenofovir disoproxil fumarate (TDF), and Tenofovir alafenamide fumarate (TAF). ETV, TDF and TAF inhibit HBV polymerate activity, to exert antiviral effects (30, 31). NAs are orally administered, well-tolerated agents that directly inhibit HBV DNA synthesis; however, they do not affect cccDNA (28).

### 3.2 IFNs

IFNs are endogenous immune molecules with broad-spectrum antiviral effects (32). IFNs mainly include type I IFN (IFN-I), type II IFN (IFN-II), and type III IFN (IFN - III) (33). IFN-I include IFN-a, - β, - ϵ, - k, - w, etc., they are produced by nearly all mammalian cells, whereas the IFN-II family consists of a single member, IFN-γ, which plays a critical role in immune regulation and antiviral defense (34). IFN-II can only be produced by activating T cells or NK cells (34). In humans, the four types of IFN-III proteins include IFN - λ 1 (also known as interleukin -29 (IL-29)), IFN - λ 2 (IL-28A), IFN - λ 3 (IL-28B), and IFN - λ 4 (35).

The production of pathogen-associated molecular patterns (PAMPs) from microorganisms or damage-associated molecular patterns (DAMPs) by host cells are detected by pattern recognition receptors (PRRs), which triggers an antiviral response. Following viral entry into the host cell, viral nucleic acids are detected by sensor proteins, including cyclic GMP-AMP synthetase/stimulator of interferon genes (cGAS/STING), TLRs (3, 7, 8, and 9), and retinoic acid-inducible gene I/melanoma differentiation-associated protein 5 (RIG-I/MDA5), which initiate a downstream antiviral signaling cascade. Among the critical proteins synthesized during this response is IFN (36).

Currently, IFN-I, primarily IFN-α and Peg-IFN-α, are clinically approved for the treatment of CHB. IFN-α has a short serum half-life and needs to be injected subcutaneously many times a week for the treatment of CHB. Peg-IFN-α includes Peg-IFN-α-2a and Peg-IFN-α-2b, they are semi-synthetic long-acting interferons obtained by PEGylation of IFN, a process that significantly extends their serum half-life (37). This article focuses on IFN-α and Peg-IFN-α.

#### 3.2.1 The antiviral and immune regulatory mechanisms of IFN-α

IFN-α binds and signals through a heterodimer transmembrane receptor composed of interferon-α receptor I (IFNAR1) and II (IFNAR2) subunits (38). Ligation of IFNAR has been shown to activate the receptor-associated protein tyrosine kinases Janus kinase 1 (JAK1) and tyrosine kinase 2 (TYK2), thereby promoting tyrosine phosphorylation of signal transducers and activators of transcription 1(STAT1)and STAT2 (38). Finally, a transcription factor complex called IFN-stimulated gene factor 3 (ISGF3) is assembled, which consists of a p-STAT1: p-STAT2 dimer and IFN regulator 9 (IRF9). Subsequently, ISGF3 enters the nucleus and binds the IFN-stimulating response elements (ISREs), activating transcription of a wide variety of IFN-stimulated genes (ISGs) (39). In this way, IFNα/β induces the expression of hundreds of ISGs, a large number of which can induce an antiviral state within the cell (40). Beyond its primary signaling through STAT1-STAT2 heterodimers, IFN-α can also activate STAT1 homodimers and other STAT proteins, expanding its functional reach into alternative cytokine signaling pathways (38). STAT1 homodimers bind to gamma-activated sequences (GAS) to induce pro-inflammatory genes. IFN-I-activated STAT3 homodimer indirectly inhibits pro-inflammatory genes. The balance between IFNAR activation and different STAT is determined in part by the relative STAT expression level (38). In non-canonical IFN signaling, IFN can induce the expression of a set of genes independent of STATs, including mitogen-activated protein kinases (MAPKs) and phosphatidylinositol 3-kinase (PI3K), to initiate the transcription of ISGs (41).

In addition to the antiviral effect, IFN-I also plays an important role in the immune regulation of the body. IFN-I have diverse effects on innate and adaptive immune cells during infection with viruses. From the perspective of the protective effects of IFN-I, IFN-I can enhance the role of dendritic cells and monocytes, promote CD4^+^ and CD8^+^ T cell responses, enhance NK cell responses, and enhance B cell responses, plays an important role in host defense against viral infections (40). IFN-I influences monocyte and macrophage function and differentiation, facilitating the transformation of monocytes into dendritic cells (DCs) with increased antigen-presenting capabilities, and modulating macrophage cytokine production, including TNF, IL-1, IL-6, IL-8, IL-12, and IL-18. This modulation enhances macrophage phagocytosis, as summarized in a review (42).

Scholars have paid attention to how IFN-I can exert immunomodulatory effects and synergistic antiviral effects in the treatment of chronic hepatitis B. Administration of Peg-IFN-α augments the suppressive impact of NK cells on regulatory T cells in chronic hepatitis B, which correlates with an enhanced HBV-specific T cell response and a notable decrease in HBsAg levels (43). Following a 15-week treatment regimen with Peg-IFN-α in humanized HBV-infected mice (huIFNAR mice), significant alterations in immune cell populations were observed *in vivo*. Human interferon-activated intrahepatic monocytes and effector memory CD8^+^ T cells are likely pivotal in the suppression of HBsAg (44). In patients undergoing treatment with Peg-IFN-α, immune control may be augmented by increasing the number of NK cells and enhancing the restoration of NK cells function (45). Peg-IFN-α modulates T cell and NK cell activity in patients on nucleos(t)ide analog therapy, enhancing T cell function and reducing HBsAg levels, particularly in those with lower baseline HBcrAg levels (46). A randomized trial demonstrated that sustained Peg-IFNα-2b therapy increased the number of CD24^+^CD38^hi^ B cells and initiated a CD24^+^CD38^hi^ B cell-centered immuno-suppressive response, which included the down-regulation of T cell and NK cell functions. This attenuated response is inversely associated with treatment efficacy, representing a key factor in the suboptimal outcomes observed with IFN therapy (47).

#### 3.2.2 IFN and clinical cure (functional cure) of CHB

Recent advancements in medical research have led to a growing interest among scholars in the clinical management of HBV, with particular emphasis on achieving a functional cure. Functional cure refers to HBsAg seroconversion even in the case of liver cccDNA persistence and cessation of liver disease. A complete cure refers to the complete elimination of cccDNA (48). Current limited courses of Peg-IFN or long-term NAs therapy can provide a sustained biochemical and virologic response, improve liver histology, and significantly reduce but not completely eliminate the risk of HCC (49). The combination of direct antiviral agents (such as NAs) and immuno-modulators (Peg-IFN) is the most effective strategy that can potentially treat the disease by integrating the effects of potent inhibition of the virus and the restoration of the host immune response (7). In clinical practice, the therapeutic efficacy of Peg-IFN combined with NAs in patients with negative HBeAg remains suboptimal. Moreover, the frequent occurrence of adverse reactions associated with IFN therapy often leads to treatment discontinuation, which subsequently impedes the achievement of optimal therapeutic outcomes.

#### 3.2.3 Common host adverse reactions in IFN therapy

Common adverse reactions of IFN-α/Peg-IFN-α include: flu-like symptoms, fatigue, mood disorders, bone marrow suppression, adult autoimmune diseases, anorexia and weight loss in children (2). In addition, adverse effects of IFN-a/Peg-IFN-α-2a in the treatment of CHB in children also include gastrointestinal upset, alopecia, thyroid dysfunction, arthralgia, rash, and growth retardation (50–52).

Fever is the most common adverse effect of IFN. Early studies, dating back to 1984, revealed that IFN induces fever by promoting the synthesis of hypothalamic prostaglandin E2 (PGE2) (53). IFN are involved in the body’s immune regulation and, as described above, IFN can activate macrophages to release cytokines such as IL-1, IL-6, and TNF-α (42). These cytokines activate brain endothelial cells in the hypothalamus, inducing cyclooxygenase-2 expression and promoting PGE2 synthesis. The resulting PGE2 then acts on thermoregulatory neurons, leading to an increase in core body temperature (54).

IFN can elicit antiviral effects via immuno-modulatory mechanisms; however, it may also contribute to the development of autoimmune diseases as a result of dysregulation within the immune system. Case reports have documented the occurrence of autoimmune diseases, such as thyroid disease (55) and systemic lupus erythematosus (56), during the treatment of CHB with IFN. A comprehensive review by Crow et al. elucidates the role of IFN-I in autoimmune diseases, with a particular focus on its involvement in the pathogenesis of systemic lupus erythematosus (SLE) and its associated complications (57). Persistent activation of the IFN-I pathway profoundly influences both innate and adaptive immunity through multiple mechanisms. It can enhance antigen-presenting cell activity, activate microglial cells, disrupt endothelial cell function, and suppresse angiogenesis. Moreover, it can induce chemokines and myeloid cell recruitment, promote apoptosis, drive B cell differentiation and immunoglobulin class switching, facilitate the expansion of T follicular helper cells, induce partial exhaustion of CD8^+^ T cells, and modulate NK cell responses. Collectively, these immune alterations contribute to progressive damage across various target organs (57).

The use of IFN therapy often leads to myelosuppresion. Type I (α and β) and Type II (γ) IFNs exert inhibitory regulatory effects on progenitor cells across all hematopoietic lineages. This includes both early (colony-forming unit-erythroid) and late (burst-forming unit-erythroid) erythroid precursors, myeloid progenitors (granulocyte-macrophage colony-forming unit), and megakaryocytic progenitors (colony-forming unit-megakaryocyte) (58). Studies have shown that IFN-α acts directly on megakaryocytic progenitor cells, inhibiting their thrombopoietin(TPO)-induced development, and IFN-α blunts the JAK/STAT signaling response induced by TPO, and IFN-α induction of suppressor of cytokine signaling 1 (SOCS-1) appears responsible for impaired TPO signaling (59). IFN-I inhibits normal hematopoietic function through the activation of p38 mitogen-activated protein kinase (60).

The neuropsychiatric adverse effects of IFN include depression, irritability, anxiety, agitation, loss of appetite, fatigue, sleep disturbance, and cognitive impairment (61). Psychiatric side effects caused by IFN-α treatment might be the result of disrupted neurogenesis or neuronal function in hippocampus (62). IFN-α activates STAT1 signaling, resulting in increased expression of ISG15, ubiquitin-specific peptidase 18 (USP18), and IL-6. This pathway contributes to reduced neurogenesis and elevated apoptosis in human hippocampal precursor cells. ISG15 and USP18 are key mediators of IFN-α-induced neurogenesis impairment and are closely associated with the observed increase in apoptosis (62). Elsewhere, it was reported that periphery-exposed IFN-α induced multiple ISGs in microglia, as well as the complement component C4b, thereby yielding genetic and phenotypic changes in microglia, and that IFN-α increased the levels of C4b modulating the aberrant synaptic pruning environment of neuropsychiatric disorders (63). IFN-α administration increased anxiety-like behaviors and decreased environmental exploration in rhesus macaques, and also enhanced the concentration of adrenocorticotropic hormone (ACTH), cortisol, and IL-6, and some monkeys exhibited depression-like shrinking behavior, which was associated with higher plasma ACTH concentrations and lower cerebrospinal fluid dopamine metabolite concentrations (64). A separate study reveals that repeated administration of IFN-α suppresses dopaminergic neuron activity. This suppression may underlie central nervous system side effects such as Parkinson’s disease-like symptoms and depression with suicidal tendencies (65). The neuropsychiatric side effects and their mechanisms are increasingly being studied.

Among the ISGs, several well-characterized proteins possess antiviral properties. These include the IFN-induced double-stranded RNA-activated protein kinase (PKR), the 2’, 5’-oligoadenylate synthetase/ribonuclease L system, adenosine deaminase acting on RNA gene (ADAR1), the Myxvirus resistance1 (Mx1) protein, inducible nitric oxide synthase (iNOS), interferon-induced transmembrane proteins (IFITMs), the interferon-induced tetratricopeptide repeat (IFIT) family, apolipoprotein B mRNA editing enzyme catalytic polypeptide-like 3 (APOBEC3, A3) family proteins, and tripartite motif (TRIM) proteins (66–68). In humans, IFN-I signaling can counteract several viral pathogens, including the influenza virus, dengue virus, herpes simplex virus, and various hepatitis viruses (39).

Li et al. performed a comprehensive overview of ISGs, which inhibit HBV and detailed the associated mechanisms, including MX2, sterile alpha motif domain-containing 4A, interferon alpha-inducible protein 6 (IFI6), TRIM14, TRIM25, ISG20, microRNA-122 (miR-122), and ADAR1, among others (67). Analysis of intrahepatic gene expression in CHB patients receiving Peg-IFN-α therapy revealed that responders exhibit marked upregulation of specific antiviral ISGs. Additionally, multiple pathways and immune cell types were identified as being closely associated with the therapeutic response to IFN-α (69). Another study highlighted that IFN can also exert antiviral effects by modulating the transcription of cccDNA, disrupting the HBV life cycle, and activating immune responses (70, 71).

## 4 Host effector proteins in IFN anti-hepatitis B virus therapy

The therapeutic effect of IFN varies among individuals. In addition to viral factors, host factors also influence the response rate of patients with CHB to IFN-α therapy. Some host factors can decrease the efficacy of IFN-α by decreasing the endogenous IFN production or interfering with IFN-related signaling pathways (72). Specific ISGs encode key effector proteins that are essential for mediating antiviral responses. A comprehensive understanding of these proteins can reveal critical mechanisms of host defense, potentially guiding the development of novel therapeutic strategies to enhance IFN efficacy, improve clinical cure rates in hepatitis B, and optimize patient outcomes (**Table 1**).

**Table 1:** Effector proteins of the host in IFN-based anti-HBV therapy.

Host effector proteins	Characterization	Antiviral function	Possible underlying mechanism	Refs
PKR	Serine/threonine kinase, activated by dsRNA-dependent autophosphorylation.	PKR can inhibit translation.	Activated PKR catalyzes phosphorylation of the translation initiation factor eIF-2α.	(73)
Mx proteins	Dynein superfamily of high-molecular-weight guanine triphosphatases, including Mx1 and Mx2.	Mx1 blocks the export of viral mRNA, interrupt s capsid formation. Mx2 decreases the levels of cccDNA and reduces HBV RNA levels by down-regulating viral RNA synthesis.	Mx1 inhibited HBV replication through a significant reduction in the synthesis of viral proteins, cytoplasmic RNAs, and DNA replicative intermediates. Mx1 binds to the HBc protein and immobilizes it near the perinuclear membrane. Mx2 reduces HBV DNAreplication by downregulating all replication markers	(80–82)
APOBEC3 family	Cytidine deaminases, comprising seven principal homologs in humans, designated as A3A through A3H.	A3 gene can inhibit HBV gene transcription and inhibit HBV viral particles. A3G interferes with the synthesis of negative-strand DNA viruses. A3G can inhibit HBV replication. A3B reduces HBsAg and HBeAg expression	The A3 gene mediates G to A-high mutations. IFN-α induces expression of A3G to bind to viral DNA polymerase. A3G can inhibit viral genomic preRNA packaging. A3B inhibits the binding of heterogeneous hnRNP K to HBV enhancer II and HBV S gene transcription;inhibits HBV S gene.	(86–90)
GBPs	A family of IFN-induced guanosine triphosphatases	GBP1 can inhibit the expression of HBs.	GBP1 inhibits its expression by directly binding to HBs.	(98)
TRIM proteins	Belongs to the RING-E3 ligase.	TRIM38 promotes protein ubiquitination and degradation. TRIM25 can degrade HBx. TRIM21 decreases HBV transcription. TRIM26 and TRIM28 can degrade HBx.	TRIM38 can up-regulate Mx1, IFIT1 and STAT1. TRIM25 promotes the ubiquitination of the HBx-k90 site. TRIM21 promotes ubiquitin-mediated degradation of hepatocyte nuclear factor 4α proteasome. TRIM26 and TRIM28 promote ubiquitin-mediated HBx degradation.	(104, 106, 107, 111, 112)
ISG20	Ribonuclease, induced by IFN-I and -II.	ISG20 degrades HBV RNA.	The C-terminal exonuclease III domain of ISG20 binds to a unique stem-loop structure called ϵ (ϵ) in all HBV RNA species.	(115)

PKR, double-stranded RNA-activated protein kinase; Mx proteins, myxovirus resistance proteins; HBV, hepatis B virus; HBc, hepatitis B core antigen; APOBEC3 family, apolipoprotein B mRNA editing enzyme catalytic polypeptide-like 3 family; GBPs, guanylate binding proteins; IFN, interferon; HBsAg, hepatitis B surface antigen; HBeAg, hepatitis B e antigen; TRIM proteins, tripartite motif proteins; RING-E3 ligase, RING finger E3-ubiquitin (RING-E3) ligase; HBx, hepatitis B x antigen; IFIT1, IFN-induced tetratricopeptide repeat 1; ISG20, interferon-stimulated gene 20.

### 4.1 PKR

PKR, a serine/threonine kinase activated through dsRNA-dependent autophosphorylation, is a critical antiviral protein induced by IFN-α (73). Activated PKR catalyzes phosphorylation of the translation initiation factor eIF-2α, resulting in inhibition of translation (74). One study indicates that PKR is a key mediator of IFN-α-induced anti-HBV activity, primarily through its role in suppressing viral protein synthesis (75). RIG-I can augment the IFN-α mediated antiviral response against HBV by upregulating the expression of PKR (76).

### 4.2 Mx proteins

Mx proteins are IFN-inducible proteins belonging to the dynein superfamily of high-molecular-weight guanine triphosphatases (GTPases), which are implicated in diverse antiviral responses (77, 78). The Mx proteins, first detected in mammals, participates in diverse cellular processes including endocytosis and mitochondria, plastid, and peroxisome dynamics (79). The expression of human Mx1 and Mx2, leads to the formation of antiviral proteins (MxA and MxB), respectively, which are well-known ISG products (80). Two mechanisms by which Mx1 inhibits HBV have been proposed. First, Mx1 blocks the export of viral mRNA (81). Second, Mx1 binds to the HBc protein and immobilizes it near the perinuclear membrane, interrupting capsid formation (82). Wang et al. demonstrated that the MX2 functions as a critical antiviral effector, acting as a host restriction factor in HBV replication by reducing cccDNA and suppressing viral RNA synthesis. Consequently, Mx2 may represent a novel intrinsic HBV inhibitor with potential therapeutic applications (83).

### 4.3 APOBEC3 cytidine deaminases

In humans, the A3 cytidine deaminases subfamily comprises seven principal homologs, designated as A3A through A3H. Among them, A3A, A3C, and A3H possess a single cytosine deaminase (CD) domain. In contrast, A3B, A3D, A3F, and A3G contain two CD domains; however, only the CD2 domain exhibits catalytic activity. The CD1 domain, while not catalytically active, participates in the binding RNA or single-stranded DNA and facilitates the incorporation of APOBEC3 into viral capsids (84). Researchers have shown that the normal human liver expresses the mRNAs of A3B, A3C, A3F, and A3G. In primary human hepatocytes, IFN-α stimulates the expression of these cytidine deaminases, and the full-length protein A3B_L_, A3F, and A3G possess anti-HBV activities (85, 86). The A3 cytidine deaminases affect HBV replication through several mechanisms, including inducing G-to-A hypermutations, inhibiting viral gene transcription, and reducing the production of viral particles (87). These G-to-A mutations are commonly observed in HBV isolates and may significantly affect the pathogenesis of HBV (88, 89). IFN-α can interfere with the synthesis of negative-strand DNA viruses by inducing the expression of A3G and binding to viral DNA polymerases (90). A3G inhibits HBV replication by inhibiting viral genome preRNA packaging, rather than by inducing G-a hypermutations (91). The A3B protein blocks the binding of heterogeneous ribonucleoprotein K (hnRNP K) to the HBV enhancer II and HBV S gene transcription, and hnRNP can promote the expression of HBV. In addition, A3B has been found to directly inhibit the HBV S gene promoter activity (92).

### 4.4 Guanylate binding proteins

GBPs are a family of IFN-induced GTPases and are the most abundant of IFN-γ-induced immune proteins (93–95). GBP1 has been implicated in the generation of innate immunity in the host and exerts antiviral and antibacterial effects. Several studies have shown that GBP1 can inhibit hantavirus (96), swine fever virus (97), hepatitis E virus (98), H5N1 influenza virus (99). Research has shown that co-treatment with Peg-IFN-α-2b and GBP1 overexpression significantly amplifies the antiviral effect of IFN, whereas silencing GBP1 partially reverses this response, underscoring GBP1’s role as a key positive regulator of IFN-α-mediated immunity against HBV. GBP1 inhibits its expression by directly binding to HBs, and the N-terminal GTP domain of GBP1 can suppress HBs. This indicates that GBP1 can inhibit the expression of HBs to block HBV infection (100). Chen et al. performed a comprehensive analysis of the antiviral response and signal activation mediated by IFN-α isoforms, and found that IFN-α14 was the most effective isoform in inhibiting HBV covalently closed circulating DNA transcription and HBV e antigen/HBV surface antigen production, and guanylate binding protein 5 was identified as an HBV restriction factor (101). The effect of GBPs on the anti-hepatitis B virus effect of IFN deserves further exploration.

### 4.5 TRIM proteins

TRIM proteins belong to the RING finger E3-ubiquitin (RING-E3) ligase and contain over 80 members (102). TRIM family members are characterized by their conserved RBCC domain that comprises the RING (R), one or two B-boxes (B), and the coiled-coil (CC) domains (103). TRIM family members are differently expressed in mouse CD4^+^ T cell subsets, macrophages, and DCs, and are constitutively expressed via IFN-I dependent and non-dependent mechanisms (104). TRIM proteins are commonly used as E3 ubiquitin ligases and are involved in diverse biological processes to modulate the key cellular functions by enhancing protein ubiquitination and degradation. These abundant c-terminal domains allow them to bind to different substrates and exert different regulatory roles, which determines the specificity and functional diversity of TRIM (105). Extensive research has highlighted the role of TRIM proteins in antiviral responses against hepatitis B. In particular, TRIM19, TRIM38, and TRIM25 have been implicated in HBsAg clearance and improved therapeutic outcomes following peg-IFN-α treatment (106, 107). TRIM38 exerts antiviral effects as ISG, inhibiting HBV replication under the stimulation of IFN-α, while up-regulating Mx1, IFIT1 and STAT1 (106). Studies have indicated that TRIM25 interacts with HBx to promote the ubiquitination of the HBx-k90 site, promoting the HBx degradation. On the other hand, TRIM25 may serve as an adapter, enhancing RIG-I recognition of pgRNAs, which further facilitates IFN production. This suggests that TRIM25 can directly and indirectly regulate HBV replication (108). TRIM21 promotes ubiquitin-mediated degradation of hepatocyte nuclear factor 4α proteasome, resulting in decreased HBV transcription levels and inhibition of HBV replication (109). The SNP rs10838543 genotype in TRIM22 influences the response to Peg-IFN-α treatment, increasing the secretion of cytokines interferon lambda 1, C-C chemokine ligand 3 (CCL3) and CCL5 in hepatocytes by modulating the cytokine-cytokine receptor interaction signaling pathway. In addition, it was positively correlated with Peg-IFNα-induced treatment response in HBeAg-positive CHB patients (110). Transient receptor potential vanillin 2 (TRPV2) is a multimodal ion channel involved in a variety of physiological and pathological processes (111). Guo et al. reported that viral infection induced TRIM21 expression via IFN-I (via autocrine signaling) and bystander cells (via paracrine signaling) of infected cells,TRIM21 catalyzes the k48-linked ubiquitination of TRPV2 at Lys295, promoting the proteasome-dependent degradation of TRPV2 and inhibiting viral infection (112). *In vitro* studies demonstrate that IFN can promote the expression of TRIM26, which inhibits HBx by promoting ubiquitin-mediated HBx degradation. In addition, TRIM26 rs116806878 may serve as a biomarker for assessing the response to Peg-IFN-α in CHB patients (113). TRIM28 can degrade HBx and inhibit HBV replication (114). The TRIM protein family, consisting of numerous members, can promote the host antiviral defenses. Its molecular mechanisms in the context of chronic liver diseases, including hepatitis B, have been extensively reviewed in the literature (115).

### 4.6 ISG20

ISG20 is an IFN-induced antiviral exoribonuclease (116). Basal level expression of ISG20 was detected in hepatocytes and was highly up-regulated following IFN treatment, while ISG20 knockdown promoted HBV replication and weakened the IFN-mediated antiviral effects. ISG20 selectively binds to a unique stem-loop structure called ϵ (ϵ) in all HBV RNA species and degrades viral RNA to inhibit HBV replication. The C-terminal exonuclease III domain of ISG20 was found to be mediate the HBV-RNA ϵ interactions (117). Elsewhere, the ISG20 expression was implicated in the development of responses to IFN-α therapy in patients with CHB. ISG20 levels in liver tissue of nonresponders were reduced prior to IFN α therapy, and ISG20 levels were highly up-regulated after the treatment in responders (118). An *in vitro* study demonstrated that ISG20 exerted anti-HBV activity by acting as a putative repressor that binds to the HBV-enhancing II/Cp region (119).

The host proteins induced by IFN antiviral therapy are diverse. IFIT3 enhances the IFN-α effector signaling pathway by promoting STAT2 phosphorylation, and overexpression of IFIT3 enhances the expression of IFN-α -triggered ISGs, including MxA, OAS1, and PKR, significantly. Decreased the secretion of HBsAg and HBeAg and the replication of HBV (120). In addition, RNA helicase DEAD-Box Helicase 5 (DDX5) has been identified as a critical regulator of STAT1 translation, with its knockdown significantly impairing the antiviral efficacy of IFN-α in inhibiting HBV replication. These findings suggest that DDX5 is a pivotal regulator in innate immunity and serves as an important factor in enhancing the therapeutic effects of adjuvant IFN-α treatment (121). Furthermore, IFI6 was found to inhibit the HBV replication in cell and mouse model by downregulating the expression of the gene of HBV enhancer II and core promoter (EnhII/Cp) (122). Other host factors, including TNF-α, mammalian p38 MAPK, IFITMs, USP18, SOCS proteins, were reviewed elsewhere (72).

## 5 Metabolism disorder and IFN antiviral therapy

Beyond the critical role of host-derived effector proteins in the antiviral action of IFN, accumulating evidence suggests that metabolism disorder can also significantly modulate the efficacy of antiviral therapies. In **Table 2**, we summarize the effects of some common metabolism disorder on IFN antiviral therapy, and explore the potential mechanisms and new ideas for improving the antiviral effect.

**Table 2:** Effects of metabolism disorder in IFN antiviral therapy and its underlying mechanisms.

Source	Metabolic type	Metabolites	Impact and possible underlying mechanisms	Refs
Host	Lipid metabolism	Triglycerides	High triglycerides can induce hepatocyte steatosis, inhibits HBsAg and HBV DNA secretion by inducing hepatocyte internal reticulum stress. Steatohepatitis due to intrahepatic lipid deposition has a negative effect on ISG expression and macrophage gene characteristics in the liver;may affect antiviral immune pathways, viral replication, and inflammatory responses. IFN-α can induce hepatic steatosis and promote a decrease in hepatitis B antigen by increasing triglyceride levels, the mechanism is not elucidated.	(124, 125, 127)
	BAs metabolism	TCA	TCA promotes HBV replication by impairing the effector function of CD^8+^ T and NK cells in CHB patients. BAs, mainly TCA reduces the number and proportion of CD^3+^CD^8+^ T cells and NK cells, inhibits the response to Peg-IFN-α treatment in HBeAg-positive CHB patients. FXR-IRF3 interaction impairs the transcriptional activity of IRF3 and impairs the activation of INF signaling and the antiviral response of cells.	(133, 134, 137)
Non-host	GM	/	Gut bacterial products may affect peripheral immunity, exert profibrotic effects, and affect the prognosis of patients with HBV-CLD.	(143)

IFN, interferon; HBsAg, hepatitis B surface antigen; HBV, hepatitis B virus; ISG, interferon stimulated gene; BAs, bile acids; TCA, taurocholic acid; CHB, chronic hepatitis B; HBeAg, hepatitis B e antigen; FXR-IRF3, farnesoid X receptor for bile acids- IFN regulator 3; GM, gut microbiota; HBV-CLD, chronic hepatitis B virus infection-associated liver diseases.

### 5.1 Lipid metabolism

Nonalcoholic fatty liver disease (NAFLD) is a common chronic liver disease that affects approximately 32.4% of the global population and leads to liver fibrosis and end-stage liver complications (123). NAFLD and metabolic syndrome are mutually causal. Abnormal lipid metabolism plays an important role in NAFLD. In order to standardize the diagnosis and treatment of NAFLD, in 2020, the International Expert Group on Fatty Liver recommended that NAFLD be renamed metabolic dysfunction-associated fatty liver disease (MAFLD) (124).Hepatic steatosis can progress to metabolic dysfunction-associated steatohepatitis (MASH), elevating the risk of cirrhosis and HCC. Within 10 years, 10-29% of MASH patients develop cirrhosis, of which 4-27% progress to HCC (125). The number of patients with hepatitis B and MAFLD has been increasing yearly, and the impact of fatty liver on the progression of hepatitis B and antiviral efficacy has received widespread investigation. Liu et al. studied the effect and mechanism of hepatocyte steatosis on HBV replication and secretion, and the results showed that hepatocyte steatosis inhibited HBsAg and HBV DNA secretion by inducing hepatocyte internal reticulum stress (126). Liver biopsy findings indicate that MASH negatively impacts ISG expression and macrophage gene signatures in the liver, potentially disrupting antiviral immune pathways, enhancing viral replication, and amplifying inflammatory responses, thereby increasing the risk of advanced fibrosis in patients with CHB (127). Findings from a prior study showed that concurrent hepatic steatosis enhanced the HBsAg response in CHB patients without overt liver failure receiving antiviral therapy, particularly in HBeAg-negative patients. Factors such as young age, those treated with NAs in combination with Peg-IFN-α, lower initial HBsAg levels, and the presence of hepatic steatosis predict the success of treatment (128). Wu et al. demonstrated that elevated lipid levels may improve HBsAg clearance in patients with CHB. IFN-α can increase triglyceride levels and induce hepatic steatosis by upregulating the expression level of acyl-CoA synthetase long chain family member 1, which indirectly promotes HBsAg clearance in patients with CHB (129). In a prospective study, patients with inactive HBsAg<200 IU/ml HBsAg were divided into treatment and control groups, and patients in the treatment group were administered with Peg-IFN for 96 weeks, and those in the control group did not receive any antiviral therapy and were observed for 96 weeks. The results showed that the cumulative clearance rates of HBsAg in patients with mild, moderate and severe steatosis at week 96 were 71.42%, 74.07% and 47.05%, respectively. The inactive HBsAg carriers with moderate steatosis exhibited the highest sensitivity to Peg-IFN, and the HBsAg clearance rate in the moderate steatosis group was significantly higher than that in the non-steatosis group (130). However, another study suggested that hepatic steatosis did not affect the virologic response, but did affect the biochemical response in patients with CHB after 48 weeks of Peg-IFN-α treatment (131). Data has shown that hepatic steatosis contributes to Peg-IFN-α therapy failure in patients with CHB (132). However, other scholars have found that mild hepatic steatosis does not influence of efficiency of 48 weeks of Peg-IFNα-2a therapy in CHB patients (133). Abnormal lipid metabolism and its associated liver disease combined with CHB are very common in clinical practice, and the traditional view is that hepatic steatosis aggravates the progression of CHB. Through this literature review, we have found different conclusions, so whether lipid metabolism and its related liver diseases occur differently under specific conditions still need further clinical observation, which should attract our attention and may become a hot spot for future research.

### 5.2 Bile acids metabolism

Bile acids (BAs) are amphiphilic molecules generated from cholesterol metabolism, and are produced in the liver and secreted into the duodenum. Studies have reported that BAs promote the digestion and absorption of lipids, regulate the metabolism of cholesterol, and enhancing bile secretion (134). Abnormal accumulation of BAs, especially taurocholic acid (TCA), stimulates the HBV replication by impairing the effector function of CD8^+^ T and NK cells in CHB patients. Notably, TCA-mediated activation of hepatic stellate cells has been demonstrated to be positively associated with cirrhosis progression. Aberrant accumulation of BAs, particularly TCA, drives macrophage polarization and creates an immuno-suppressive tumor microenvironment that promotes initial tumor expansion and tumor growth in HCC (135). Zhen et al. found that serum BAs, especially TCA, were significantly elevated in HBeAg-positive CHB patients compared with those in healthy individuals and patients in other phases of chronic HBV infection. Moreover, serum BAs, particularly TCA, can block the response to Peg-IFN-α treatment in HBeAg-positive CHB patients. Mechanistic investigations have shown that in the HBeAg-positive CHB patients, BAs reduced the number and proportion of CD3^+^CD8^+^ T cells and NK cells, and impaired effector function (136). Others have uncovered that BAs promote the transcription and expression of HBV genes in hepatocyte lines, and BAs stimulate the HBV gene expression to abolish the antiviral effects of IFN-α (137). However, evidence shows that BAs synthesis is enhanced in different cells, including immune and parenchymal cells, following viral infection, suggesting that BAs participate in the initiation of cellular antiviral responses (138). Liang et al. showed that the farnesoid X receptor (FXR)–interferon regulatory factor 3 (IRF3) interaction impairs the transcriptional activity of IRF3 and damages the activation of interferon signaling and the antiviral response of cells. FXR inhibitors have been shown to enhance IFN expression and promote cellular antiviral responses (139). Notably, BAs function as endogenous agonists of mitochondrial protein 2 (MFN2), and physiologically relevant levels of BAs may strengthen the innate immune response by facilitating MFN2-mediated pathogen detection. However, if the host fails to clear the invading pathogens, resulting in liver damage and cholestasis, elevated BA levels may shift toward amplifying excessive inflammatory responses. Although this may initially serve as an adaptive defense mechanism, it can ultimately exert detrimental effects on the host (140).

### 5.3 Gut Microbiota

The liver receives intestinal content via the portal system, biliary tract, and systemic circulation. The symbiotic relationship between GM and liver is regulated and stabilized by a complex network of interactions, which include metabolic, immunological, and neuroendocrine dialogues between them (141). GM can directly interact with viral particles and affect their infectivity (142). Furthermore, gut microbiota can initiate and activate host antiviral immunity (143). A review by Wirusanti et al. summarized the underlying mechanisms of GM-IFN -virus interactions: 1. GM can promote steady-state IFN-I expression from macrophages and plasmacytoid dendritic cells (pDCs) and homeostatic IFN-III expression from intestinal epithelial cells. 2. Gut microbiota-derived PRRs ligands stimulate the expression levels of IFN. The components of the symbiotic GM generate molecular patterns that can bind to PRRs, and this pattern recognition activates downstream signaling pathways and IFN-I production. 3. Gut microbiota-derived metabolites activate IFN expression (144). *In vitro* studies have shown that exposure of peripheral blood mononuclear cells to fecal-derived bacterial extracts in patients with hepatitis B without cirrhosis promoted the expansion of T helper 17 cells, while exposure to BE in patients with cirrhosis decreased the number of TH1 cells, suggesting that peripheral immunity may be an integral part of the mechanisms by which the overall bacterial product exerts a profibrotic effect and influences the prognosis of chronic hepatitis B virus infection-associated liver diseases patients (145). Another animal study found that the immune tolerance pathway of HBV was activated in young mice at 6 weeks of age before the establishment of gut bacteria. Moreover, the maturation of gut microbiota in adult mice stimulated hepatic immunity via the TLR4-dependent pathway, leading to rapid clearance of HBV (146).

## 6 Other host factors associated with IFN antiviral therapy

The effectiveness of IFN-based antiviral therapy is influenced by a wide range of host factors. Parameters such as age, ALT levels, and gender have been extensively studied for their impact on IFN response in hepatitis B treatment, and thus will not be reiterated here (72). The role of other small molecules, single nucleotide polymorphisms (SNPs), cytokines, and chemokines (CXCLs) in antiviral therapy still needs further research (**Table 3**).

**Table 3:** Effects of other factors in IFN antiviral therapy and its underlying mechanisms.

Other host factors	Characterization	Name	Function and possible underlying mechanisms	Refs
miRNAs	miRNAs are small, non-coding RNA molecules.	MiR27b-3p MiR-548c-3p MiR-3613-3p MiR-6126	In IFN-α treatment, exosomes with declined miR-27b-3p triggered activation of RIG-I/TBK1 signalling in macrophages against HBV. MiR-548c-3p targets TRIM22 to attenuate the therapeutic effect of Peg-IFN-α. MiR-3613-3p impairs the IFN-induced immune response by targeting CMPK1 in CHB. *In vitro* assays revealed that miR-6126 was able to suppress HBsAg production and HBV replication and the mechanism is still unclear.	(149, 150, 152, 153)
SNPs	/	The G alleles of rs2278420 and rs6509607 The rs7519753 C allele PNPLA3 SNP (rs738409)and TLL1 SNP(rs17047200)	The G allele of rs2278420 and rs6509607 were associated with mRNA level of ZNF350, with an increased probability of Peg-IFNα response in HBeAg-positive CHB patients, likely through the modulation of JAK-STAT signaling pathway. The rs7519753 C allele was strongly associated with higher hepatic TP53BP2 expression.TP53BP2 can promote JAK/STAT signaling by downregulating SOCS2. PNPLA3 SNP was associated with hepatic steatosis. In Peg-IFN therapy, PNPLA3 SNP and TLL1 SNP were related to the treatment efficacy, patients without minor alleles of these SNPs had a high virologic response and significant reduction in their HBsAg titer.	(154–156)

IFN, interferon; MiRNA, microRNA; RIG-I, retinoic acid-inducible gene I; TBK1, tank-binding kinase 1; TRIM22, tripartite motif 22 protein; Peg-IFN-α, polyethylene glycol-interferon-α; CMPK1, cytidine monophosphate kinase 1; CHB, chronic hepatitis B; HBsAg, hepatitis B surface antigen; SNPs, single nucleotide polymorphisms; ZNF350, zinc finger protein 350; TLL1, tolloid-like 1; JAK/STAT, Janus kinase/signal transducers and activators of transcription; SOCS2, suppressor of cytokine signaling 2.

### 6.1 MicroRNAs

MiRNAs are small, non-coding RNA involved in the regulation of gene expression by binding to the complementary sequences of the 3’-untranslated region of the target mRNA. This interaction promotes mRNA degradation or translational inhibition, mediated by RNA-induced silencing complex. MiRNAs are involved in various biological processes such as cell development, differentiation, proliferation, apoptosis, and metabolism, and play a role in the pathogenesis of inflammation, fibrosis, and carcinogenesis in liver diseases (147, 148). The mechanisms of miRNAs regulate IFN-I signaling transduction include influencing cell surface IFNAR expression, the activity of STATs, and ISGs expression (41). It has been shown that there is a correlation between serum miRNA levels and IFN treatment response in patients with CHB. Nagura et al. confirmed that serum miR-192-5p levels were associated with IFN treatment response in CHB patients, and multivariate analysis showed that lower miR-192-5p levels at baseline were independent predictors of virologic response (149). Elsewhere, it was found that peripheral circulating exosomes-miRNAs influenced sensitivity to IFN therapy in patients with CHB (150). Moreover, there is evidence that exosomes with low concentrations of miR27b-3p in the serum of Peg-IFN-α treatment responders activate interferon regulator 3/7 in the macrophage IFN-α synthesis pathway (IRF3/7). However, over-expression of miR27b-3p in HepAD38 cells inhibits IFN-α synthesis in macrophages, decreasing the capacity to eliminate HBV, while this inhibition can be alleviated by the use of inhibitors released by exosomes (151). miR-548c-3p also targets TRIM22 to attenuate the therapeutic effect of Peg-IFN-α in HBeAg-positive patients with CHB (152). Pretreated plasma miR-301a-3p and miR-145-5p levels were higher in CHB patients who achieved a collective response or HBsAg loss after Peg-IFN/NA treatment compared with non-responding patients (153). Zhao et al. showed that miR-3613-3p impairs the IFN-induced immune response by targeting cytidine monophosphate kinase 1 in CHB (154). Elevated serum miR-6126 levels during Peg-IFN therapy were associated with a 1-log reduction in HBsAg 48 weeks post-treatment. *In vitro* studies further demonstrated that miR-6126 suppresses HBsAg production and HBV replication (155).

### 6.2 SNPs

Recent studies have begun to uncover the role of SNPs in host genes in influencing individual differences in HBV infection susceptibility and IFN response (156). There is a research indicating that the G alleles of Rs2278420 and Rs6509607 are associated with the mRNA levels of zinc finger protein 350 (ZNF350), which may increase the probability of Peg-IFN-α response in HBeAg-positive CHB patients by regulating the JAK-STAT signaling pathway. Low levels of ZNF350 mRNA are associated with complete response, with higher levels of ZNF350 mRNA in patients with suboptimal response. However, it has not yet been fully explained how SNPs regulate the IFN-α signaling pathway (156). Guan et al. found a new SNP (rs7519753) by genome-wide association study (GWAS), the rs7519753 C allele was significantly associated with serum HBsAg loss after Peg-IFN-α treatment in CHB patients, and TP53BP2 expression is higher in liver tissues of individuals carrying this allele. TP53BP2 is a nearby gene located 18 kb downstream of SNP rs7519753, which can promote JAK/STAT signaling by down-regulating SOCS2, thereby enhancing the response of liver cells to IFN - α *in vitro* and *in vivo* (157). In a study examining the effects of NAFLD-associated SNPs in patients with HBV infection, only patatin-like phospholipase domain-containing protein 3 (PNPLA3) SNP was significantly associated with hepatic steatosis. In Peg-IFN therapy, PNPLA3 SNP and TLL1 SNP influenced the therapeutic efficacy, and patients without minor alleles of these SNPs showed good results with high virologic response and significantly reduced HBsAg titers (158). In conclusion, future studies about host SNPs will help to identify the advantageous personnel who use Peg-IFN-α/IFN-α to treat CHB, improve the efficiency of treatment, and avoid economic losses caused by ineffective treatment.

### 6.3 Other factors

Furthermore, the expression levels of molecules, including cytokines, chemokines, IFITs, and IFITMs, can be used to assess the efficacy of IFN in the treatment of CHB. In a study conducted by Wang et al., IFN-related gene chips were utilized to analyze patients with CHB undergoing IFN therapy. The findings indicate that high baseline expression levels of CXCL10, IFIT1, and IFITM1 may serve as predictive biomarkers for a favorable response to IFN therapy. High expression of interleukin-13 receptor α 1 subunit gene, IL15, IFI35 and IFI44 molecules, and decreased expression of IFN-associated growth regulator 2, IL11 receptor α subunit, interleukin 4 receptor, IFN regulator 3, IRF4, Pyrin and HIS domain family member 1 and ADAR molecules may indicate poor IFN response in patients (159).

IL-17A pretreatment decreased the levels of ISGF3 complex and antiviral-related ISGs (ISG15, ISG20 and Mx1) in resonse to IFN-α stimulation, increased the expression of suppressor of cytokine signaling (SOCS) 1, SOCS3 and USP18 (160). Peg-IFN-α therapy upregulate the expression levels of IFN-γ, IL-12p70, IL-7, IL-8, IL-16, IL-4, an IL-13 among responders prior to the observable decline in HBsAg. Furthermore, the levels of several chemokines, including CCL11 (Eotaxin), CCL24 (Eotaxin-2), CCL26 (Eotaxin-3), CCL4 (MIP-1β), CXCL1 (GRO-α), CXCL9 (MIG), CXCL11 (I-TAC), CXCL12 (SDF-1α), and CXCL13 (BLC), were also found to be elevated in these responders (161). Further investigations are warranted to clarify the roles of these cytokines and chemokines in the treatment of CHB with IFN.

## 7 Conclusion

Inflammatory and even neoplastic diseases of the liver caused by HBV infection aggravate the prognosis of patients infected with hepatitis B. However, combined with the characteristics of HBV and the current treatment status, there are still great challenges in the cure of hepatitis B. Peg-IFN-α remains the most promising clinical option for achieving a cure in CHB. Beyond the previously discussed host factors, several viral factors also play a significant role in modulating the treatment’s efficacy. Due to individual differences, not all patients are candidates for IFN antiviral therapy, and there are some adverse reactions during IFN therapy that cause treatment to be discontinued.

Several novel antiviral drugs are being investigated in clinical trials. However, as indicated in the article, no new antiviral drugs have yet received approval for use. Among the drugs under development, GSK3228836 (also known as GSK836 or Bepirovirsen) has attracted significant attention and has entered phase III clinical trials. GSK836 is a free-form antisense oligonucleotide specifically engineered to suppress viral protein expression by leveraging gene silencing mechanisms. Data from Phase I and II clinical studies indicate that GSK836 is safe, effective, and well-tolerated, showing great potential to reduce HBsAg levels and inhibit HBV DNA replication (162). In a multicenter study, weekly administration of 300 mg bepirovirsen for 24 weeks resulted in a 9 to 10% rate of sustained HBsAg and HBV DNA loss, maintained for 24 weeks after treatment discontinuation. The results were comparable between participants that received NA therapy and those that did not receive such treatment (163).

Nevertheless, monotherapy does not achieve a high rate of HBsAg clearance, which calls for the adoption of multi-drug combinations for better outcome. The optimal regimen for such combination therapy needs to be further established. Furthermore, strategies to sustain HBsAg clearance and minimize recurrence following the discontinuation of treatment after achieving clinical cure require further investigation. Developing a thorough understanding of host-related antiviral mechanisms is essential. Future research should focus on amplifying factors that enhance interferon therapy, suppressing elements that reduce its effectiveness, and investigating combination therapies to minimize adverse effects. In future, researchers should investigate whether drug combinations can significantly improve patient prognosis of hepatitis B.

## Appendix

**Table:**

Abbreviations	Expanded form
ADAR	Adenosine deaminase-acting RNA
AHB	Acute hepatitis B
APOBEC3	Apolipoprotein B mrna editing enzyme catalytic polypeptide-like 3
ACTH	Adrenocorticotropic hormone
BAs	Bile acids
CAM	Capsid assembly modulators
cccDNA	Covalently closed circular DNA
CCL3	C-C chemokine ligand 3
cGAS/STING	Cyclic GMP-AMP synthetase/stimulator of interferon genes
CHB	Chronic hepatitis B
CXCL	Chemokines
CXCLs	Chemokines
DAMPs	Damage-associated molecular patterns
DCs	Dendritic cells
DDX5	Dead-box helicase 5
EGFR	Epidermal growth factor receptor
ETV	Entecavir
GBPs	Guanylate binding proteins
GM	Gut microbiota
GTPases	Guanine triphosphatases
HBcAg	Hepatitis B core antigen
HBeAg	Hepatitis B e antigen
HBsAg	Hepatitis B surface antigen
HBV	Chronic hepatitis B virus
HBx	Hepatitis B x
HCC	Hepatocellular carcinoma
HSPG	Heparan sulfate proteoglycans
IFI6	Interferon alpha-inducible protein 6
IFIT family	Interferon-induced tetratricopeptide repeat family
IFITMs	Interferon-induced transmembrane proteins
IFN - III	Type III IFN (IFN - III)
IFN-I	Type I IFN
IFN-II	Type II IFN
IFN-α	Interferon-α
IFNAR	Interferon-α receptor
IL	Interleukin
iNOS	Inducible nitric oxide synthase
IRF9	IFN regulator 9
ISGF3	IFN-stimulating gene factor 3
ISGs	IFN-stimulating genes
JAK	Protein tyrosine kinases Janus kinase
MAFLD	Metabolic dysfunction-associated fatty liver disease
MAPKs	Mitogen-activated protein kinases
MASH	Metabolic dysfunction-associated steatohepatitis
MFN2	Mitochondrial protein 2
Mx proteins	Myxvirus resistance proteins
NAFLD	Nonalcoholic fatty liver disease
NAs	Nucleos(t)ide analogs
NK cells	Natural killer cells
NRP1	Neuropilin-1
NTCP	Sodium taurocholate cotransporter peptide
PAMPs	Pathogen-associated molecular patterns
Peg-IFN-α	Pegylated interferon α
PGE2	Hypothalamic prostaglandin E2
pgRNA	Pregenomic RNA
pgRNA	Pregenomic RNA
PKR	IFN-induced double-stranded RNA-activated protein kinase
PNPLA3	Patatin-like phospholipase domain-containing protein 3
PRRs	Pattern recognition receptors
rcDNA	Relaxed circular DNA
RIG-I/MDA5	Retinoic acid-inducible gene I/melanoma differentiation-associated protein 5
RING-E3	RING finger E3-ubiquitin
SNPs	Single nucleotide polymorphisms
SOCS	Suppressor of cytokine signaling
STAT	Signal transducers and activators of transcription
TAF	Tenofovir alafenamide fumarate
TCA	Taurocholic acid
TDF	Tenofovir disoproxil fumarate
TLR	Toll-like receptor
TNF-α	Tumor necrosis factor-alpha
TPO	Thrombopoietin
TRIM proteins	Tripartite motif proteins
TYK2	Tyrosine kinase
TRPV2	Transient receptor potential vanillin 2
USP18	Ubiquitin-specific peptidase 18
WHO	World health organization
ZNF350	Zinc finger protein 350
